# An exploration of how nurse prescribing is being used for patients with respiratory conditions across the east of England

**DOI:** 10.1186/1472-6963-14-27

**Published:** 2014-01-21

**Authors:** Nicola Carey, Karen Stenner, Molly Courtenay

**Affiliations:** 1 School of Health and Social Care, University of Surrey, Surrey, England

**Keywords:** Access, Adherence, Nurse prescribing, Respiratory disease, Risk management, Vulnerable groups

## Abstract

**Background:**

There is a need to reduce symptoms, exacerbations and improve quality of life for patients with respiratory diseases. Across the world, increasing numbers of nurses are adopting the prescribing role and can potentially enhance service provision. Evidence suggests improved quality of care and efficiencies occur when nurses adopt the prescribing role. No evidence is available on the views of nurse prescribers who care for respiratory patients. The aim was to explore how nurse prescribing is being used for patients with respiratory conditions in different care settings across one strategic health authority, and whether this has benefited patients, healthcare professionals and the National Health Service.

**Methods:**

A qualitative study involving semi-structured interviews with a purposive sample of 40 nurses who prescribed for respiratory patients across the six counties in the East of England Strategic Health Authority. Data were collected in 2011 and subject to thematic analysis.

**Results:**

Disease management, including treatment and prevention of exacerbations, emergency episodes and minor illness, optimising and co-ordinating care were key aspects of care provided. Findings are reported under three themes: access, adherence and risk management and impact on nurses. Prescribing enabled nurses overcome existing problems in service provision to improve access, efficiency and patient convenience, reducing hospital admissions and length of stay. It also enabled patient centered consultations, which encouraged self-management, improved adherence, helped manage expectations, and reduced inappropriate service use. While participants experienced increased job satisfaction, knowledge and confidence, concerns were raised about increased responsibility, support, governance and future commissioning of services in line with planned major changes to the National Health Service.

**Conclusions:**

This study provides new knowledge about how nurse prescribers provide care to patients with respiratory diseases. Despite a lack of consensus over the most effective model of respiratory care, prescribing was reported to have improved and extended points of access to treatment, and supported management of complex patients, particularly vulnerable groups. Given the high burden of chronic respiratory disease to patients and families this has important implications that need to be considered by those responsible for commissioning services in the United Kingdom and other countries.

## Background

Chronic disease (CD), the main reason why people seek healthcare in the developed world [1-3], encompasses a set of progressive and long-term medical conditions including respiratory diseases such as asthma and chronic obstructive pulmonary disease (COPD) [2,4,5]. Around 300 million individuals are affected by asthma [6] and 10% of adults aged over 40 years have COPD across the world [7,8]. Asthma and COPD are both treatable conditions, the aim of which, whatever the severity, is to minimize and manage symptoms (GINA 2011, GOLD 2010). Despite this, many patients experience high levels of symptoms, poor quality of life, and exacerbations that are a common cause of emergency hospital admissions [9-11].

Each year in the United Kingdom (UK), £286 million is spent on 110,000 admissions for COPD [10,12], and £61 million on 80,000 admissions for acute asthma attacks [11]. In addition to hospital admissions, there are a number of other significant direct costs (e.g. medication and on-going management), and indirect costs (e.g. time lost from work or school or premature death) associated with the management of these diseases (GINA 2011, GOLD 2010).

Traditional healthcare systems, largely built around an acute, episodic model of care, are ill-equipped to meet the requirements of those with conditions such as asthma and COPD [5,13,14]. Many countries are responding to the global problem of changing demands on healthcare by seeking to improve primary care services in order to reduce hospital admissions for people with long term conditions (LTC) [13,15]. In order to meet this changing requirement the contribution of non-medical health professionals, particularly nurses, has increased in recent years [15,16]. It is recognized that nurses have lead roles to play in the delivery of services to patients with chronic respiratory diseases (CRD) such as asthma and COPD [17], and when nurses incorporate advanced roles in to their practice quality of care, access to services, and patient satisfaction improve [18-20].

Multidisciplinary working, that integrates clinicians from primary and acute care services, is considered important in ensuring the provision of a comprehensive respiratory service [9,21]. However, there is a lack of consensus in the evidence as to the best strategy for providing care [17,22,23]. A qualitative study in the UK, found that planning of respiratory care services was inconsistent, dependent upon local decision making by organisations, and focused on a small number of high risk people with complex needs [9,22].

In the UK, the pyramid of care for LTCs has been used to differentiate risk levels of patients so that patients at high risk of hospital admission can be targeted for case management [24]. This has coincided with the introduction of new roles, such as community matron (CM) and case managers. Although these roles have a specific remit to reduce hospital admissions [25,26], evidence of success is lacking [14,23,27]. At the same time, as part of policy to improve access to healthcare there has been an evolution of nurse-led immediate-access services (such as walk-in centres (WIC), minor illness clinics and out-of hours services (OOH)) [28]. Consequently, a broad range of nurses in both primary and secondary care, including specialist nurses, practice nurses and nurse practitioners, now encounter patients with acute and on-going respiratory conditions [21,22].

Expanding nurses’ roles to include prescribing, is a key component of the UK governments’ National Health Service (NHS) modernization agenda [29-32]. In contrast to other countries, e.g. the United States, Australia and Canada, in the UK nurse prescribing is not restricted to advanced nurse practitioners [33-35]. Evidence suggests that the majority of the 26,000 Nurse Independent Prescribers (NIP), who have virtually the same prescribing rights as doctors [36], are based in primary care, with an increasing number taking on the role in secondary care [37,38] (see below overview of non-medical prescribing (NMP) in the UK).

### An overview of non-medical prescribing in the UK

Non-medical Prescribing (NMP)

A generic term used to describe healthcare professionals e.g. nurses, pharmacists and allied health professionals (radiographers, podiatrists, and physiotherapists) and optometrists who have the legal capacity to prescribe medicines using independent or supplementary prescribing once they have undergone a formal programme of preparation.

**Nurse Independent Supplementary Prescribers:** first level registered nurses who complete an educational programme (27 taught days and 12 days in practice) are awarded dual qualification.

a) Nurse independent prescribing (NIP)

May assess, diagnose and prescribe independently any licensed or unlicensed drug, except some controlled drugs for treatment of addiction within area of competence [29,31,39,40]

b) Nurse supplementary prescribing (NSP)

•Form of dependent prescribing. Initial diagnosis made by doctors, clinical management plan (agreed by Dr, patient and NSP) details medicines that can be prescribed within area of competence [30,32]

Other healthcare professionals

•Pharmacists, Physiotherapists and Podiatrists, can train as independent and supplementary prescribers [30,32]

•Optometrists, and allied health professionals (i.e. radiographers) able to train as supplementary prescribers [30]

Findings from two recent UK surveys designed to explore the therapy areas in which nurses prescribe, indicate that around 35% contribute to the care of patients with respiratory conditions [38,41]. Additional evidence from the United States suggests Advanced Nurse Practitioners practicing in respiratory care frequently prescribe medicines for the management of acute and chronic respiratory conditions [42], however international evidence exploring the prescribing practices of nurses who care for patients with respiratory conditions is lacking [33,35].

Although not specific to patients with respiratory conditions, research in other CD areas, such as diabetes, suggests that a number of benefits arise when nurses adopt the prescribing role. Patients report that they like the continuity of care, comprehensive information and holistic care that they receive [43,44], whereas nurses report improved access to medicines and cost savings achieved through improved efficiency [45,46]. A recent audit of 2,500 episodes of prescribing across 15 NHS trusts in the north west of England reported that nurse prescribing (NP) averted substantial numbers of general practice (GP) and consultant appointments, reduced episodes of unscheduled care, reduced length of hospital stay, prevented readmissions and identified numerous cases of inappropriate prescribing [47].

Although nurses in general are positive about the adoption of the role as prescriber [37] there are on-going concerns surrounding inconsistent levels of support and governance structures [48,49]. Barriers to nurse prescribing have been reported including restrictions of local arrangements, such as inability to computer generate prescriptions, and organisational and policy restrictions [38,49].

No evidence is currently available on the views of nurses who care for respiratory patients on the adoption of prescribing in their practice or service provision. This is important given the number of patients with CRD who experience high levels of symptoms, poor quality of life, and exacerbations and the high number of nurses working in a variety of roles who prescribe medicines in this area of practice.

### Aim

The aim of the study was to explore how nurse prescribing is used for patients with respiratory conditions in different care settings across one strategic health authority (SHA). The main objective was to explore if and how local developments in nurse prescribing have benefited patients, healthcare professionals and the NHS.

At the time of the study, the NHS in England was divided in to 10 areas and managed by SHAs. Each SHA had the responsibility to manage healthcare organisations (including primary care trusts, acute trusts, mental health trusts and GPs) across a large geographical area.

## Methods

### Design and Setting

In this paper we report on a set of interview data that formed the third and final stage of a study that explored how non-medical prescribing (NMP) (see overview of NMP in UK) is used across the East of England (EOE) SHA. In stage 3, a qualitative approach, using semi-structured telephone interviews, was used to explore the views of nurses who cared for patients with respiratory conditions. Findings from phase 1 of this study, NMP leads views on their role and the implementation of NMP from a multi-organisational perspective, and phase 2, a survey of NMPs across the EOE SHA have previously been reported [38,48].

### Participants

Participants were purposively selected from 138 respondents in the aforementioned questionnaire [38] who reported that they prescribed for patients with respiratory conditions, and indicated that they would be interested in taking part in further research. An information sheet and invitation email, with a reminder two weeks later, was sent to all eligible participants (all nurses) who provided contact details. Those interested (32%) contacted the researcher to arrange an interview. A total of 40 interviews with nurse prescribers were conducted, four additional interviews were planned but not undertaken as staff were unavailable during dates arranged for interviews. Consent was obtained before each interview and procedures to protect confidentiality i.e. removing identifiable information from the transcripts explained.

### Data collection

Semi-structured telephone interviews were conducted and audio recorded by two experienced qualitative researchers (NC, KS). The interview schedule was informed by the literature [33,37,48] and findings from phase 2, a survey of NMPs across the EOE SHA [38]. The interview schedule covered background information about nurse prescribers’ role and practice setting, scope of practice, general views and experience of prescribing for patients with respiratory conditions, difficulties arising from prescribing, support and supervision. Interviews, lasting 40-60 minutes, were held at mutually convenient times. Data collection took place from September to November 2011.

### Data analysis and rigor

A thematic analysis, a recognised method used to identify, analyse and report themes and patterns within interview data was conducted [50]. Atlas Ti, a qualitative data analysis software package, was used to aid initial coding and identification of themes. This was followed by further discussion and interpretation between two researchers (NC & KS) to identify areas of data convergence and overall interpretation of the themes. Saturation of the data was achieved. Once themes were developed the data was analysed by job title to identify any differences. Member validation occurred in that an overview of the findings was emailed to all participants in March 2013, who agreed and acknowledged a high level of relevance in the reported findings.

### Ethical consideration

Ethical approval for the study was provided by the University of Surrey. The study was deemed a service evaluation by Cambridgeshire Research Ethics Committee.

## Results

### Demographic details

Participants (n = 40) were from all six counties in the EOE, and worked across a wide range of practice settings (i.e. GP, hospital in and out patients, community, WICs, OOHs and the prison setting), with 75.5% (n = 31) based in primary care, and 32.5% (n = 13) working as GP nurses (see Table 1). There was considerable variation (range 4-100%) in the amount of time participants spent with respiratory patients. Respiratory nurse specialists had a dedicated role whereas it constituted only a small part of the role of others who worked in WICs and or provided minor illness services in GP.

**Table 1:** Participant Information

***n = 40***	***n = number of participants***	**% of total sample**
**Job Title**		
**General practice based nurses** (nurse practitioner, practice nurse, practice nurse lead)	13	32.5
**Community matrons** (Long term condition case manager)	7	17.5
**Paediatric nurses** (nurse specialist, nurse practitioner, community children’s nurse)	6	15.0
**Secondary care nurses** (emergency nurse practitioner, critical care outreach, nurse consultant)	4	10.0
**Hospital or mixed base respiratory nurse specialists**	4	10.0
**Community based respiratory nurses** (lead nurses, specialist nurse, nurse consultant)	3	7.5
**Other non-hospital based nurse practitioners** (out of hours, walk in centre, prison)	3	7.5
**Geographical location**		
Cambridgeshire	11	27.5
Essex	10	25
Suffolk	10	25
Bedfordshire	4	10
Norfolk	4	10
Hertfordshire	1	2.5
**Employer**		
Primary care trust (including community trust, community mental health & provider services)	16	40.0
General practice	12	30.0
Acute	9	22.5
Other: walk in centre, out of hours, social enterprise	3	7.5
**Setting**		
Primary care: community, walk in centre, out of hours, prison	15	37.5
Primary care: general practice	12	30.0
Secondary care	9	22.5
Primary & secondary care	4	10.0
**Age range**		
25-35 yrs	2	5.0
36-45 yrs	7	17.5
46-54 yrs	21	52.5
55-64 yrs	4	10.0
**Years qualified as a prescriber**		
1-2 years	3	7.5
3-5 years	16	40.0
> 5 years	20	50.0
**Use of prescribing qualification**		
Independent prescribing	37	92.5
Supplementary prescribing	1	2.5
Independent & supplementary prescribing	1	2.5
Not prescribing	1	2.5
**Specialist respiratory training**		
Yes (diploma, degree and/or masters level module, study days, other accredited learning)	34	85.0
No	6	15.0
**Time spent on respiratory patients**		
Range 4-100%, Mean = 51%, Mode = 100%		

There was similar variation in the frequency of prescribing (range 2 to 50 times per week), which, in line with the number of presentations of upper respiratory tract infections and chest infections, tended to rise in the winter months. Ninety five per cent (n = 38) used NIP to prescribe a range of medicines e.g. bronchodilators, mucolytics and steroids, anti-puritics, analgesia, and antibiotics, inhaler devices, and nebulisers: a few participants were also involved in providing oxygen therapy.

Participants described how managing CRD, including the treatment and prevention of exacerbations, emergency episodes of respiratory care (no previous diagnosis of CRD), minor illness and optimising and co-ordinating care were key aspects of care provided (see overview of respiratory care provided by NMPs).

### Respiratory care provided by non-medical prescribers

Type of care episode

•Treating exacerbations [previous diagnosis of CRD] (n = 35)

•Preventing exacerbations of CRD (n = 34)

•Minor respiratory illness (n = 21)

•Routine monitoring (n = 17)

•Optimise care within specialist respiratory service (n = 8)

•Unscheduled episodes of respiratory care [no previous diagnosis of LTC] (n = 5)

•Co-ordinate care (n = 5)

Diagnosis & scope of practice

•Diagnose exacerbations of CRD (n = 33)

•Treat pre-diagnosed respiratory condition (n = 19)

•Initial diagnosis of respiratory condition (n = 12)

•Diagnose acute respiratory condition [no previous diagnosis of CRD] (n = 8)

Patient management

•Advice re self-management and education (n = 37)

•Initiate respiratory medicines (n = 33)

•Repeat prescribing (n = 20)

•Prescribe for co-morbidities (n = 17)

•Make recommendation(via patient notes, telephone, letter or email for medicine(s) to be prescribed) (n = 14)

•*(CRD = chronic respiratory diseases i.e. asthma or chronic obstructive pulmonary disease)*

#### Themes

Three themes 1) access, 2) adherence and risk management and 3) impact on nurses, explore the challenges faced, and impact when prescribing for patients with respiratory conditions.

Quotations are used to illustrate themes. To protect anonymity of participants, references to names or places have been removed from these quotations. Names have been replaced by an 'x’ where appropriate. Job titles have been abbreviated to NC = nurse consultant, CRNS = community respiratory nurse specialist, RNS = respiratory nurse specialist, CM = community matron/case manager, NPr = Nurse practitioner, ENP = emergency nurse practitioner.

### Access

By prescribing, participants reported that they were able to improve access to medicines, convenience for patients and enhance service efficiency. They were able to overcome problems with existing services in relation to a) frail and housebound patients, b) gaps in routine care, and c) access to treatment in hospital. Rapid detection and treatment of acute episodes or exacerbations of respiratory conditions was a care priority for all participants and was one of the most significant areas to which prescribing contributed (see above overview of respiratory care provided by NMPs).

### Frail and housebound patients

Many patients had multiple co-morbidities with complex health, particularly mental health, and social care needs. For these reasons, home visits were predominantly used by CMs and CRNS to provide care, alongside nurse-led community clinics. Being able to prescribe in the community was reported to increase access to medicines, sometimes by days. This was said to contribute to faster recovery and reduction in hospital admissions.

“the biggest thing for patients is the delay in getting prescriptions, especially when they’re acutely unwell. People that are not prescribers have to come back at the end of the day, write a letter on a computer, fax it over to the GP, check that they’ve got that fax, the GP needs to read and agree with it and then a prescription gets generated. Sometimes it takes up to 2 days, whereas if I’ve gone to see them I can prescribe it there and then.” [p30, NC]

In order to maximise efficiency, preventative prescribing was often used for patients at risk of developing acute exacerbations. This meant that some participants in GP and the community issued advanced prescriptions for antibiotics and/or steroids which, in the event of an infection, could be used under the advice of the nurse. Importantly, the immediacy of access to treatment reduced anxiety and panic symptoms as patients were more confident that treatment was in hand.

“They can get the drug when they want it. Thinking of exacerbations, this time of year, the patient’s main fear is that they can’t get a GP’s appointment quickly enough when they’re feeling unwell. Even if the GP is happy to issue antibiotics and steroids because you’re a known COPD, it can sometimes take 24-48 hours for that to be delivered to them, and then they’re starting to get into 'panic mode’ and their breathing gets worse.” [p29, CRNS]

Gaining agreement to prescribe for patients attached to different GPs, remote access to patient records, and electronic prescribing facilities were key challenges facing nurses who wished to prescribe for patients in the community. While improvements were underway, nurses who prescribed for patients in their home were unable to electronically prescribe and few had access to electronic patient records. Incomplete referral information meant nurses had to take a more detailed history, and or contact the GP, all of which was time consuming.

“What we get on the referral is what we know. I think we’ve had three more practices now go on to the same system we’re on and the GPs are finally coming round to understanding that sharing their notes is a benefit to all of us. So it is improving. I’ve now got two [GP practices] on my caseload where I can see their notes as well.” [p28, NC]

### Gaps in routine care

Poor attendance for routine care was reported to be common for patients with CRD. By prescribing, nurses extended the settings in which patients could access a complete package of care and was fundamental to providing routine care for those who would not normally access it.

“If they said that we could no longer prescribe, that would have a huge impact, especially on our client group. And I would question that people probably wouldn’t use our services if that they knew that we couldn’t prescribe.” [p19, NPr]

Prescribing also enabled adaptations to services. In place of disease specific clinics, for example, some participants offered a single 'one-stop-shop’ review appointment for patients with multiple LTCs. NP was felt to improve the overall experience as it reduced waiting times and travel costs by eliminating the need for patients to return to the practice setting to collect a prescription, or see a GP.

“I can do their prescription there and then, whereas sometimes they’d have to come back for it. For the younger people, who have taken time off work, they don’t want to come back again, and sometimes they get angry or frustrated if it puts them out, so yes, it’s much, much better for them that it’s done there and then.” [p20, children’s community nurse]

Within immediate access services (such as WIC, OOH, prison and minor illness clinics in GP) NPs offered same-day appointments and/or telephone advice. Here, having an NP on the team, expanded the type of care provided and enabled provision beyond historical ways of working (e.g. patient group directives). Participants believed that compared to doctors they were more accessible and available for consultations. Patients were able to access medication and commence treatment sooner, reducing the likelihood of worsening symptoms and or complications related to the respiratory condition.

“We pick things up earlier because they feel that they can come and see us sooner than they would normally go to a doctor, because they don’t want to bother a doctor. So I think us doing prescribing and monitoring and treating chest conditions means that patients present earlier, they get less complications.” [p8, NPr]

A more controversial outcome was that some patients used immediate access services for repeat medicines that should have been obtained through GP.

“My main dealings are treating people with acute respiratory problems. Their medicines’ (ran) out, or they’re becoming ill with complications. That’s mainly an out of hours setting. It is a benefit for them to walk in to the walk-in centre. At least they’re getting care somewhere.” [p15, NPr]

### Access to treatment in hospital

Within hospital, the length of time between assessment and treatment was traditionally dependent upon access to a doctor. Through NP, patients attending hospital (as inpatients, outpatients or Accident and Emergency (A&E)) were said to receive treatment more rapidly. The speed and efficiency of NP was said to improve recovery and contribute to reducing hospital stay, the number of GP consultations and visits to A&E.

“It’s had an enormous difference to patient care. So if they come to clinic and they’re exacerbating, we’re able to act upon it straight away. It actually stops them needing an appointment with the GP to start the medication. It has prevented hospital admissions, repeat consultations with the GPs and patients coming to A&E because of delays in treatment.” [p31, RNS]

Again, as a result of wider policy changes such as the European working time directive (i.e. legislative changes in 2004 which restricted the work hours of junior doctors in the National Health Service and other healthcare systems throughout Europe), certain services, such as overnight critical care outreach, had become reliant on the presence of a NP.

“Junior doctor hours have been cut quite dramatically, especially the hospital at night and so at, 3 o’clock in the morning there’s just not the doctors around, we are relied upon to see a lot of patients which normally would be seen by the medical staff.” [p6, critical care outreach]

### Adherence and risk management

#### Adherence

A common problem noted by participants was that many patients used their medicines incorrectly. Poor adherence was thought to be caused by a lack of understanding, unsuitable prescribing and/or a lack of a self-management plan.

“If a child has an asthma exacerbation……when they come to us [hospital clinic], they sometimes don’t have an asthma plan, and they don’t know that they can multi-dose, they think that 2 puffs is the maximum, so that’s quite suspect because they could reduce the amount of admissions they have and their anxiety if they knew how to give things properly.” [p39, paediatric NPr]

Providing education about self-management of asthma and COPD was a crucial part of the care provided by all participants. Key aspects included education related to general asthma management, ensuring patients had good inhaler technique, checking medication regimens, checking patient adherence to regimens, developing self-management plans, and smoking cessation advice. Specialist respiratory nurses were also involved in optimising treatment, particularly for complex patients with multiple conditions. The application of specialist prescribing knowledge was said to enhance these processes.

#### Managing co-morbidities

Co-morbidity was described as common in this group of patients and prescribing was described as challenging as it required knowledge and understanding of potential drug interactions and contraindications across a range of conditions.

“A lot of our patients now, they’re on several disease registers so they’ve actually got lots of things to manage and managing medicines and the interactions and the patients in front of you is quite difficult, it’s very complicated.” [p7, NPr]

Building the confidence and skills required to undertake complex decisions required support. There was concern over situations where such support was not readily available, particularly where specialist advice may be required.

“[The prescribing qualification] makes you wary, especially when you’re giving an inhaled corticosteroid or prednisolone to a diabetic and you get all the contraindications and red alerts. To begin with, that was really scary, but with having support of my manager and everything on the risks and benefits, it’s all about weighing up those things, but over time you get better at doing it.” [p29, RNS]

#### Prescribing and the patient consultation

Across all settings, by enabling nurses to complete episodes of care and provide a more seamless service, prescribing produced subtle changes in the nature and content of the consultation. For example, patients received medicines alongside the explanation from the nurse on how to use them, and were more likely to receive the intended treatment. This was thought to improve consistency and adherence.

“It’s much more personal, you can go more in-depth, where before I could prescribe it was a case of, “Right, OK, I think you’ve got a chest infection”, and then they’d perhaps have another appointment to come and see someone who could just double check and then give them medication as well, I’m like a one stop shop now.**” **[P8, NPr]

In addition, prescribing was felt to contribute to more holistic and patient centred care, partly due to NPs high level of knowledge and experience in this area of practice. Nurses were able to work closely with the patient to explain treatment and develop a personalised plan of care. This in turn encouraged patients to become more actively involved in decisions about the management of their treatment. These factors were said to be beneficial in preventing exacerbations and also for providing routine monitoring of patients with CRD.

“The consultations are patient-centred, hopefully, so obviously you can meet with the patient and the patient can tell you what their agenda is and obviously you can have a list and then form a plan together.” [p18, NPr]

Within the hospital setting, being able to complete the episode of care was felt to offer a more consistent standard of care, particularly in areas such as A&E where doctors regularly changed as part of a rotation.

“If you are a prescriber working alongside a doctor who is a locum, you will be guiding them to best local practice or best evidence-based practice, because it’s your department that you work in. So I think that patients are getting a more consistent standard of care across the department, so less variability for regular conditions that the department sees.” [p3, ENP]

For those working in the community, seeing patients in their home environment helped tailor advice and treatment to the person. This was due to a number of factors; patients were thought to be more honest and talkative at home, meeting family members or carers was valuable for sharing information, and the nurse could observe aspects of lifestyle that patients did not disclose, such as the presence of pets or stockpiling medicines.

“When you go through the medication with them and they’ll go to a cupboard and it’s full, you can pick up very quickly if people are stockpiling or not taking things, but don’t want to admit it because they’re worried the doctor’s going to be cross. I’m in a much better position to stop things or go back to the patient and explain why they should be taking it.” [p25, CM]

#### Managing emergency or preventative medicine

Despite the benefits of prescribing, the causes of poor adherence were complex and not easy to resolve. Providing an accurate assessment of patients with CRD was sometimes difficult to achieve where patients under-reported the severity of their condition.

“I think there’s under-reporting of symptoms in some respects and because with some respiratory conditions they’re very variable it’s actually quite difficult to make a full assessment at times.” [p11, clinical lead]

Use of emergency services for what was considered routine care raised concerns about the potential misuse of medicines, in particular, preventative prescribing of steroids and antibiotics. Patients were said to overuse these medicines, even when they did not have the symptoms of an infection, because they immediately reduced unpleasant symptoms of breathlessness and wheezing.

“One thing that often goes hand in hand respiratory disorders is an anxiety disorder. So what you find is you have your steroids and your antibiotics in the cupboard and you’ve got all these lovely instructions on when to use them. If their anxiety kicks off, which then makes them short of breath and feel very het-up, they will use, as a crutch, steroids and antibiotics.” [p24, CM]

Education about long-term side effects did not always prevent misuse. It was recognised that social isolation and anxiety further complicated the situation and could heighten symptoms. Calling patients for review rather than simply issuing repeat prescriptions was one way of managing this risk, although non-attendance was a problem. Other precautions included undertaking risk assessments, restricting the number of repeat prescriptions or requiring permission from a doctor for preventative medicines.

“We risk-assess people that we’re going to leave medications with, especially steroids. I think we’ve had one or two that have actually abused it because they feel so good when they’re on it. They come off of it and then… and that’s all about education, saying when you come off of this you’ll actually go back to this level.” [p29, CRNS]

Managing expectations was felt to reduce inappropriate use of services. Nurse specialists would explain to patients that they would only prescribe for respiratory conditions. In comparison, community nurses would prescribe repeat medicines for other conditions, such as diabetes. This was noted as being potentially problematic because without appropriate follow-up, there was the possibility of missing underlying issues around adherence.

“I have to judge each visit as it goes by the amount of stress I feel is going on in the home, but it is actually counter-productive in the end because I can’t actually monitor their level of usage. The reason they may have run out may not be because they’re forgetful but it might be because there’s poor compliance, or over-use of medications.” [p21, CM]

### Impact on Nurses

#### Job satisfaction, knowledge and confidence

Across all settings, the increased autonomy associated with being able complete an episode of care through prescribing gave participants more job satisfaction, status, and respect from patients and other healthcare colleagues.

“I think it’s been a marvelous thing really and it’s been good, it’s good for my confidence, it’s given me a lot to think about. It’s given me a new string to a bow, it, keeps me interested.” [p6, critical care outreach]

Prescribing was reported to increase knowledge and confidence, maximise skills and enable participants to take responsibility for decisions about patient management. Nurses described how their learning accelerated once they began to prescribe and took on the responsibility of a more autonomous role.

“My knowledge is much better which means that I’m much better at assessing patients. I’m much better at giving advice to patients because I’ve got more detailed knowledge about those drugs and how they work and how they interact and it does allow for opportunistic kind of advice for people who are non-compliant in medication. It’s made me a much better and more rounded nurse practitioner.” [p3, ENP]

Prescribing was seen as essential to many roles, including community matrons, case managers and respiratory nurse specialists:

“It makes a huge difference - there are some CMs and case managers in our area that can’t prescribe and they’re all desperate to get the qualification now because once you start the job, you don’t realise what an impact it has on your job until you’re doing it.” [p24, CM]

#### Anxieties and concerns

With increased responsibility came increased anxiety related to the potential for making mistakes or being penalized for prescribing decisions. Poor access to medical notes was a concern (mainly in community and out-of-hours settings). Developing competence to manage co-morbidities, expand scope of practice and prescribe new medications were challenging areas for some nurses. The extent to which nurses took on new areas of practice varied and depended on access to training, exposure to experience and support. While anxiety was greatest during the initial period after qualifying, confidence grew over time.

“I think the actual prescribing, you sort of grow into it as you learn after the course. When I started, I prescribed very, very few things, and hardly at all, and over time I’ve gradually sort of grown in, into it as I’ve learned more.” [p10, NPr]

Numerous concerns were raised about the future commissioning, support and governance of NMP. A number of health trusts had recently undergone structural reorganisation which had resulted in gaps in the provision of support and leadership for NMP. Different attitudes of new trust managers to, for example, accepting funding from pharmaceutical companies for educational events, had reduced provision of NMP support. In some cases, trusts that previously supported an NMP lead, NMP forum and educational updates had been assimilated into a new trust that now provided only minimal support.

There were fears that within this climate of change, where GPs and managers sought greater control over stretched resources, roles were at stake and that the value of NMP in contributing towards efficiency savings may not be recognised. Concerns were also raised that in the future, trusts and GPs would employ nurse prescribers on lower grades with less experience, opt for nurses to use patient group directions, and or restrict what nurses can prescribe.

“I’m not sure my role will exist in the future. The GP practice want their own case manager but paid at a lower grade, someone who will prescribe, but only want to pay a band six to, not a band seven, and there are people with my prescribing skills at band five, so they’ll definitely get someone. One of my colleagues she’s actually limited in what she can prescribe because they [as a GP] decide what they will and won’t prescribe.” [p26, CM]

## Discussion

This is the first study to explore prescribing by nurses for patients with respiratory conditions.

Unlike areas such as diabetes, where nursing roles have been explored in some detail [51,52], there is little equivalent information in respiratory care against which to compare those who prescribe [17,21]. The findings reveal that nurses across a wide spectrum of roles, titles, and settings, prescribe for patients with respiratory conditions. The variety of nursing roles in this SHA may reflect different patterns of local service provision for respiratory diseases. Inconsistent strategic planning for respiratory services was noted by Pinnock *et al.* (2009); a situation that is not helped by the lack of consensus over the most effective model for respiratory care [21,23].

Participants’ experience and contact with respiratory patients varied, as did scope of practice and level of decision-making. While the majority used prescribing skills to manage exacerbations, provide education and advice on self-management, there was considerable variation in the extent that individuals were involved in routine monitoring, the initial diagnosis of a respiratory condition, initiating new medication, diagnosing and treating minor illness and prescribing for co-morbidities. Variations in the level of autonomy practiced by respiratory nurses in primary care have previously been reported by Upton *et al. *[17]. Similarly, the extent that nurse prescribers review patients independently of a medical professional and are responsible for patient management has been shown to vary [37,53]. While it is evident that prescribing practice will be influenced by service requirements, role variation, and skills mix within teams, our results indicate that nurse prescribers are increasingly working independently.

Despite variations in practice, the impact of prescribing was reported as beneficial by participants in our study, to the extent that the success of services had become dependent upon nurses’ ability to prescribe. The findings add to the body of evidence that NP is meeting its anticipated aim of improving access to medicines [29]. Across all settings, participants reported improved access, particularly amongst the more vulnerable. A key area to benefit was the prevention and management of acute exacerbations in high risk patients. This perhaps provides some evidence that NP contributes to a reduction in hospital admissions and length of stay, as shown in a recent audit in the north west of England [47]. In contrast, the findings of studies designed to explore nurse-led interventions on hospital admission (such as case management), have had mixed results [23]. Similarly, empirical work designed to articulate the impact of the nurse prescribing role on patient outcomes (such as a reduction in hospital admissions) is also lacking; further evaluative research in this area is therefore required.

In addition to improving access to medicines, benefits reported by participants in our study included more appropriate advice and treatment, continuity of care, and improved personalised care. These findings align with those reported previously about nurse prescribing for other LTCs [43,44]. Patients with LTCs are said to be more satisfied with, and value, nurses who can prescribe and practice independently whilst maintaining a person-centred approach [19]. Our findings indicate that adoption of the prescribing role by nurses improved the quality of medicines advice and the promotion of self-management. These benefits of NP are in-line with the aims of LTC models which advocate personalised care planning [54].

There is evidence that patients with severe and complex LTCs benefit less from educational self-management interventions [23]. Our findings suggest NP can help with the management of patients with complex physical and social problems. For example, nurses who visited patients in their own home reported that they were able to make more accurate holistic assessments based on a deeper insight into personal or lifestyle factors, which in turn, improved the effectiveness of prescribing decisions. In addition, providing faster and more effective treatment through prescribing was considered to impact on patients’ emotional health and reduced feelings of anxiety and panic that can escalate respiratory conditions. This is important given that the high burden of CRD, in terms of quality of life, to patients, families and carers [12].

Prescribing in community settings did, however, present a set of challenges. It was evident from our findings that nurses had to be successful at building good relationships with GPs in order to gain agreement to prescribe across different practices. Funding arrangements and agreements were not always in place, creating potential inequalities in service provision. Poor IT infrastructures in community settings hampered access to patient records and electronic prescribing. These findings support those reported by Lupari *et al*. [14] in a recent review of the literature, that compared to other practice settings, fewer governance systems and support for on-going training and supervision are available for NPs in community settings.

Shortfalls have previously been identified in the training of respiratory nurses in primary care: 20% of nurses working in an advanced role (but not necessarily prescribing) caring for patients with asthma, and 52% caring for patients with COPD, were reported not to have obtained accredited training [17]. Whilst the level of specialist training was high in our sample, there was a general concern amongst participants about the level of support available to prescribe new medicines and for patients with co-morbidities. Despite this, prescribing facilitated changes to the organisation of care for patients, particularly those in GP, including the introduction of generic LTC clinics and, single review appointments for patients with multiple LTCs. Patients with multiple LTCs are reported to dislike the disjointed care and less personalised consultation style they sometimes receive in disease specific clinics and would prefer to have all their conditions considered in one appointment [19]. Managing patients with complex conditions and co-morbidities requires a high level of knowledge, decision-making and autonomy that many NPs find challenging. In a survey by Latter *et al. *[37]*,* 58% of NIPs reported that they had concerns about prescribing for patients who have co-morbidities. This is an area that requires more attention to ascertain how best to support NPs to develop complex decision-making skills.

In line with Pinnock *et al.’s *[9] findings, there was some indication of a need to improve routine care. Despite the documented effectiveness and availability of self-management plans, evidence suggests that they are not routinely given out or used, and that non-use is strongly associated with poor control [55]. Hamilton *et al. *[22] argue that it important to provide a comprehensive service across all areas of care rather than focus on the short-term gains of reducing hospital admissions. They add that some patients experience difficulty navigating the complex care systems and wanted more flexibility over access [56]. In our study, the points at which patients could access treatment had been extended through nurses prescribing in different settings. A perhaps unintended consequence of this was that some patients wanted routine medication prescribed in these new settings, such as walk-in-centres. There is a risk that potential misuse of medication or misunderstandings about medicines regimes may not be picked up when patients access routine care outside of GP. In addition there may be issues with lack of continuity of care and lack of access to full medical history. The dilemma facing nurses in this situation is whether these risks outweigh the benefits of providing immediate treatment. We identified a number of strategies that nurses used to assess and manage risk in these situations alongside those related to prescribing of preventative treatment for exacerbations. Our findings suggest that although GP has historically been the preferred setting for provision of routine care [21], a more flexible approach is required. New technologies, such as telephone reviews, and use of mobile technology as Worth *et al.* (2011) suggests offer innovative approaches to this aspect of care. For this to be successfully achieved, fundamental changes to IT infrastructure, access to medical records and local prescribing arrangements would need to be addressed.

### Limitations

As a qualitative study, we acknowledge that the findings reflect the views of participants from one SHA who volunteered to be interviewed. It should be considered that organisational arrangements for respiratory care and nurse prescribing may vary across different geographical regions and as such may not represent the experiences of NPs working in this practice area. There is, however, no single model for respiratory care in the UK or elsewhere. We are confident that the sample included enough variation in participant roles and settings to provide a comprehensive picture of nurse prescribing in this practice area. There are however, gaps in this picture with respect to district nurses and health visitors, school nurses and other non-medical prescribers such as pharmacists, and physiotherapists who were not part of our sample. In order that the views of patients with respiratory disease can be evaluated and understood further research, using different methodologies, is required.

## Conclusion

This study provides new knowledge about how nurse prescribers provide care to patients with respiratory diseases. Despite a lack of consensus over the most effective model of respiratory care, prescribing was reported to have improved and extended points of access to treatment, and supported management of complex patients, particularly vulnerable groups. Given the high burden of chronic respiratory disease to patients and families this has important implications that need to be considered by those responsible for commissioning services in the UK and other countries around the world.

## Competing interest

No conflict of interest has been declared by the authors.

## Authors’ contributions

NC and MC were involved in study design, NC and KS in data collection and analysis, NC, KS, and MC in manuscript preparation. All authors read and approved the final manuscript.

## Authors’ information

Joint authors: Nicola Carey and Karen Stenner.

## Pre-publication history

The pre-publication history for this paper can be accessed here:

http://www.biomedcentral.com/1472-6963/14/27/prepub
